# “Locked in a cage”—A case of dengue virus 4 encephalitis

**DOI:** 10.1371/journal.pntd.0005369

**Published:** 2017-05-04

**Authors:** Deborah H. L. Ng, Sapna P. Sadarangani

**Affiliations:** 1 Department of Infectious Diseases, Institute of Infectious Diseases and Epidemiology, Tan Tock Seng Hospital, Singapore, Singapore; 2 Lee Kong Chian School of Medicine, Nanyang Technological University, Singapore, Singapore; Centre Hospitalier Universitaire de la Réunion, RÉUNION

## Case presentation

A 62-year-old Chinese woman was admitted to the hospital with a four-day history of fever associated with chills, rigors, and headache. She also complained of postural giddiness, five episodes of vomiting, and shortness of breath on exertion for one day. She denied any abdominal pain or bleeding manifestations. Her past medical history was unremarkable, and she had not travelled out of Singapore.

Clinical evaluation revealed tympanic temperature of 38.2°C, blood pressure of 140/80 mmHg, and pulse rate of 92 beats per minute. The patient was alert and orientated with a Glasgow Coma Score (GCS) of 15. She had a faint maculopapular rash, but physical examination was otherwise unremarkable; the lungs were clear to auscultation, and there was no abdominal tenderness or hepatomegaly. Hematology and biochemistry results on admission are summarised in Table 1. Chest radiograph was normal, with no radiographic evidence of pleural effusion. Serum dengue NS1 antigen was positive, and dengue IgM and IgG were negative (SD BIOLINE Dengue Duo Cassette, Alere).

**Table 1:** Hematological and biochemical markers.

Investigation	Day of illness					
	4	5	6	7	10	19 (at follow-up visit)
White blood cell, x 10^9^/L (range 3.6–9.3)	1.9	4.0	4.1	6.4	7.3	
Neutrophil, x 10^9^/L (range 1.40–5.90)	1.2	1.32	1.8	4.6	4.8	
Hemoglobin, g/dL (range 11.0–15.0)	13.0	13.2	13.9	13.8	12.4	
Hematocrit, % (range 35–45)	39.5	39.8	42.2	42.6	38.4	
Platelets, x 10^9^/L (range 170–420)	106	62	91	123	220	
Creatinine, μmol/L (range 40–75)	60	60	50	91	69	
Alanine aminotransferase, U/L (range 14–54)	66		218	148	146	76
Aspartate aminotransferase, U/L (range 15–41)	85		155	73	76	25

During the initial two days of her admission, the patient did not develop any further dengue warning signs, such as abdominal pain, hepatomegaly, persistent vomiting, or bleeding manifestations. Her platelet count nadir was 62 x 10^9^/L (day 5 of illness). She developed confusion and receptive and expressive aphasia with inability to recognize her family members on day 6 of illness. On examination, she was afebrile, GCS was E4V2M5, and there were no signs of meningism. Apart from the receptive and expressive aphasia, a full neurological examination did not reveal any motor or sensory deficit. A computed tomography scan of the brain that day showed no acute cerebral infarct or hemorrhage. Her hematocrit had risen by 6.8% but did not fulfil criteria for significant hemoconcentration; thrombocytopenia was mild, and although transaminitis was worse, it rapidly improved (shown in Table 1). Workup for nosocomial infection comprising blood cultures and urinalysis was unrevealing. Cerebrospinal fluid (CSF) analysis revealed a nucleated cell count of 62 cells/uL (97% lymphocytes, 2% macrophages, 1% neutrophils), an erythrocyte count of 9 cells/uL, a protein count of 2.33 g/L, and a glucose count of 3.5 mmol/L (serum glucose 5.9 mmol/L). CSF Gram stain and culture, tetraplex polymerase chain (PCR) testing for Cytomegalovirus, herpes simplex virus (HSV), varicella zoster virus (VZV), and *Toxoplasma gondii* were negative. CSF enterovirus PCR testing was negative, as were tests for CSF measles and mumps serology. We proceeded to do magnetic resonance imaging (MRI) of the brain and angiography with contrast the next day (day 7 of illness and day 2 of confusion) due to the persistent confusion with aphasia. There was no evidence of restricted diffusion or abnormal susceptibility, with no focal abnormal signals in the cerebrum, brainstem, or cerebellum. There was also no abnormal parenchymal or leptomeningeal enhancement seen on the contrasted images. Diffusion-weighted imaging with apparent diffusion coefficient mapping scans were normal, with no MRI evidence of blood–brain barrier leakage, and there was no hydrocephalus, midline shift, or effacement of basal cisterns.

Serum dengue PCR (on day 4 of illness) using an in-house protocol adapted from the Centers for Disease Control DENV-1-4 Real-Time PCR Assay for detection and serotype identification of dengue virus (DENV) was positive for DENV-4. CSF dengue PCR (using the same methodology) was negative on day 6 of illness. NS1 antigen from the CSF was negative, while both dengue IgM and IgG were positive (SD BIOLINE Dengue Duo Cassette, Alere).

Although the patient was afebrile at the onset of confusion, there was recrudescence of low-grade fever within 24 hours, which persisted until day 7 of illness. She was empirically treated with intravenous acyclovir until the VZV and HSV PCR results were available. By day 8 of illness, she was able to verbalise and recognise family members but still not orientated as to the time and place. In view of her improved mentation, electroencephalography (EEG) was not performed, although nonconvulsive seizures should be a consideration of altered mentation in patients with suspected encephalitis. She made a complete clinical recovery at day 10 of illness. Repeat clinical examination when she was lucid and cooperative revealed no symptoms or signs suggesting peripheral nerve, spinal cord, or ocular involvement. At follow-up, she had no neurological sequelae. She recollected seeing herself “locked in a cage,” trying to get out on the day she had developed confusion, but was unable to recall any other details regarding the episode of confusion or aphasia. She reported no other psychotic symptoms.

## Case discussion

### Dengue encephalitis

Although dengue virus is not classically known to be a neurotropic virus, it can cause encephalopathy and other neurological manifestations, such as encephalitis, myelitis, and Guillain-Barré syndrome. [1] Encephalitis is an increasingly recognized entity, with reports of 4%–47% of patients admitted with an encephalitis-like illness in endemic areas. [2]

The case definition for dengue encephalitis as proposed by Soares [3] includes fever, acute signs of cerebral involvement, positive IgM dengue antibody or dengue PCR on serum and/or cerebrospinal fluid, and exclusion of other causes of viral encephalitis and encephalopathy, as demonstrated in our patient. The recent statement published by the International Encephalitis Consortium suggests that the case definition for encephalitis includes the major criteria of altered mental status (such as diminished or altered level of consciousness, lethargy, or personality change) lasting ≥24 hours, with no alternative cause identified and with at least two other minor criteria present. Minor criteria include the following: documented fever ≥38°C within 72 hours of presentation (either before or after), generalized or partial seizures not completely attributable to a known seizure disorder, new onset of focal neurologic findings, CSF pleocytosis (CSF white blood cell [WBC] count ≥5/mm^3^), new or acute brain parenchymal abnormalities on neuroimaging suggesting encephalitis, and, finally, EEG abnormalities consistent with encephalitis not attributable to another cause. [4] Our patient’s presentation supports the diagnosis of confirmed or probable encephalitis, with altered mental status and consistent febrile illness, focal neurological findings of confusion, aphasia, and the sensation of being “locked in a cage.” In addition, she also had compatible CSF pleocytosis, although her neuroimaging was normal.

The pathogenesis of dengue encephalitis is not well understood. Direct viral neural invasion and altered blood–brain barrier permeability are possible mechanisms for dengue encephalitis occurring during the acute dengue illness. The role of host immune response is not well defined. A case series of ten patients with neurologic manifestations of dengue (three with myelitis, five with Guillain-Barré syndrome, one with optic neuromyelitis, and one with a case of encephalitis) found that seven patients had positive dengue IgM and nine had positive dengue IgG in the CSF. [5] The authors also evaluated intrathecal synthesis of antibodies in the CSF using an antibody index requiring comparison of quantitative dengue-specific antibodies in CSF and serum, with dengue IgG antibody index of ≥1.5 defined as suggestive of intrathecal antibody synthesis. Only the three myelitis cases in the case series (out of nine patients who had positive CSF IgG) had evidence of intrathecal antibody synthesis based on the definition. The single encephalitis case did not fulfill the antibody index criteria but had both positive CSF dengue IgM and IgG at day 7 of illness, i.e., a similar time frame as our patient. Although our patient’s CSF dengue PCR was negative at day 6 of dengue illness (day 1 of confusion), a report of a fatal case of DENV-4 encephalitis demonstrated positive dengue PCR in the CSF on day 3 of illness. [6] In addition, a case report of fatal dengue hemorrhagic fever reported the presence of dengue virus in histopathological specimens of brain tissue. [7] There is insufficient evidence from published literature to make definitive conclusions regarding time kinetics of the presence of dengue virus in CSF and viral load in patients with probable/confirmed dengue encephalitis and that relationship with the appearance of dengue-specific antibodies in the CSF. Although the post-dengue immune-mediated syndromes (e.g., myelitis and Guillain-Barré syndrome) that occur well after recovery of acute dengue infection are well described, the host immune response may play a contributory role in pathogenesis of dengue encephalitis occurring during acute dengue illness.

Our patient had encephalitis concurrent to her acute dengue illness without any other manifestations of severe dengue; although we could not demonstrate positive CSF dengue PCR (at day 6 of illness), we demonstrated CSF pleocytosis with positive CSF dengue IgM and IgG. The reported case of dengue encephalitis by Puccioni-Sohler [5] also had positive dengue IgM in the CSF, but there was a paucity of reported clinical data to conclude that the neurological manifestation was solely an immune-mediated phenomenon. Further research, well-designed case–control studies, and deeper understanding are needed to uncover the mechanisms and pathogenesis for neurological manifestations (such as encephalitis) of dengue virus infection, including the role of host immune response. Although the IgG antibody index is not a suggested criterion for diagnosis of dengue encephalitis, its diagnostic role can be further evaluated in future studies using quantitative titers from serum and CSF (methods such as plaque reduction neutralisation test are time- and expertise-intensive assays, typically requiring specialised laboratories).

There are no reported pathognomonic features of dengue encephalitis on brain imaging. A small case series of patients with neurological manifestations of dengue (five with encephalitis and one with encephalomyelitis) reported cerebral edema on the computerized tomography (CT) or MRI brain scans of three patients. [8] There have also been reports of nonspecific changes, such as low- or high-density signals in different areas of the brain and meningeal enhancement, as well as normal scans. [9]

### Diagnosis of dengue encephalitis in areas endemic for dengue and other flaviviruses

Dengue is endemic in Singapore, with 11,298 reported cases in 2015. [10] DENV-1 and DENV-2 were the predominant circulating serotypes, and DENV-4 was the least common.

In a study of 21 patients with dengue fever presenting with neurological manifestations, ten patients had dengue virus isolated from their serum and/or CSF samples; four were positive for DENV-2, five for DENV-3, and one for DENV-1. [11] Another case series also reported dengue encephalitis with DENV-2 and DENV-3. [12]

There have been reports of co-positivity of serum dengue and Japanese encephalitis (JE) IgM in areas endemic for both infections. [13] Another study from Thailand found that 11% of CSF samples from JE patients were also positive for anti-dengue IgM. [14] JE has been rare in Singapore since pig farming was phased out in 1992, with the last case being reported in 2005. [15]

Zika virus has been reported to have a high level of cross-reactivity to dengue antibody responses. [16] However, Singapore had no known imported or locally transmitted cases of Zika virus at the time of presentation. [17] Our patient had not travelled to JE-endemic areas or received vaccination against another flavivirus (such as JE or yellow fever) and had confirmed positive serum dengue PCR and positive CSF dengue antibodies in the context of compatible clinical illness. Nonetheless, it is important to elicit a detailed travel history for areas which are hyperendemic for both dengue and JE, since co-infections have also been reported. [13]

Chikungunya, an alphavirus, can occasionally be associated with encephalitis (in particular, during the Reunion Island outbreak). [18] Our patient did not have prominent arthralgia or arthritis/synovitis to clinically support a suspicion of co-infection with Chikungunya. Furthermore, Singapore has had sporadic cases of Chikungunya since 2013, following initial importation in 2008 and outbreaks in 2013. [19] The patient had not travelled out of Singapore to areas at higher risk for chikungunya. However, co-infections should be considered in the appropriate clinicoepidemiological context.

## The presenting case

Our patient with DENV-4 infection developed new acute neurological symptoms of confusion and expressive aphasia at day 6 of her acute dengue illness. CSF showed lymphocytic pleocytosis with a positive CSF dengue IgM and IgG. Testing for other pathogens in CSF was negative. She had not travelled to areas endemic for other flaviviruses. Although DENV-4 has been reported to potentially cause encephalitis, those cases were fatal, with multiorgan involvement and dengue haemorrhagic fever. [6,7] By contrast, our patient had no other features of severe dengue, such as severe plasma leakage, bleeding, or other organ impairment. This is the first known case of DENV-4 encephalitis with complete recovery.

**Key learning points**

- Dengue encephalitis is rare, but clinicians should be aware of the diagnostic criteria and consider the diagnosis in endemic areas.
- In areas that are also endemic for other flaviviruses, co-infection needs to be differentiated from cross-reactive serology.
- Brain imaging may be helpful but does not confirm or exclude the diagnosis of dengue encephalitis by itself, and CSF analysis for dengue PCR and serology is also important.
- Pathogenesis of neurological manifestations of dengue leading to encephalitis are incompletely understood but could include direct neural invasion with intrathecal antibody synthesis or alterations in the blood–brain barrier.
